# Taxonomic notes on *Cryptamorpha sculptifrons* Reitter (Coleoptera, Silvanidae), with description of its larval morphology

**DOI:** 10.3897/zookeys.412.7615

**Published:** 2014-05-29

**Authors:** Takahiro Yoshida, Toshiya Hirowatari

**Affiliations:** 1 Entomological Laboratory, Graduate School of Bioresource and Bioenvironmental Sciences, Kyushu University, Fukuoka, 812–8581 Japan; 2 Entomological Laboratory, Faculty of Agriculture, Kyushu University, Fukuoka, 812–8581 Japan

**Keywords:** Taxonomy, redescription, lectotype, variety, urogomphi

## Abstract

*Cryptamorpha sculptifrons* Reitter, 1889 is redescribed and a lectotype and paralectotype are designated. The mature larva of *C. sculptifrons* is described. It is hypothesized that a variety described by Grouvelle (1908), *C. sculptifrons* var. *punctifrons* from India, might not be conspecific with Japanese *C. sculptifrons*. It is also suggested that larvae of *Cryptamorpha* can be distinguished from larvae of the tribe Brontini by the relatively thick antennae and the 3rd antennomere which is less than 3/4 of the length of the 2nd.

## Introduction

The family Silvanidae Kirby, 1837 (Coleoptera, Cucujoidea) includes two subfamilies, about 58 genera and approximately 500 species (Thomas and Leschen 2010) throughout the world, of which 43 species have been recorded from Japan (Hirano 2009, 2010 and Yoshida and Hirowatari 2013, 2014). Silvanidae are considered to be fairly primitive among the Cucujoidea, and most of its members seem to be fungivorous (Thomas 2002).

The genus *Cryptamorpha* Wollaston, 1854 (Brontinae, Telephanini) includes 27 described species globally (Thomas and Leschen 2010; Brown et al. 2012). In Japan, two species and one undetermined species were recorded by Hirano (2009, 2010). *Cryptamorpha desjardinsi* (Guérin-Méneville, 1844) is the only species of the subfamily Brontinae listed in the provisional list of alien species naturalized in Japan (Murakami and Washitani 2002). There is little biological information on other *Cryptamorpha* species. *Cryptamorpha sculptifrons* Reitter, 1889 was described on the basis of specimens collected by George Lewis from Japan. This species was listed in the taxonomic key of the genus *Cryptamorpha* by Grouvelle (1919), and two varieties were described by Grouvelle (1908). However, there was no figure of the male genital structure and no redescription of this species based on the type specimens.

While larval morphology can provide useful information for phylogenetic studies (e.g. Lawrence et al. 2011; Leschen et al. 2005), there is little information on the larval morphology of this family, and the larvae of *Cryptamorpha* species are poorly known.

In this paper, we redescribe *Cryptamorpha sculptifrons* and designate a lectotype and paralectotype. In addition, we describe the larval morphology of this species for the first time.

## Materials and methods

### Observation of morphology and dissection and photographic technique

External characters were observed under a stereoscopic microscope (Olympus SZX10). Genital structures were placed on a cavity slide glass with 50% glycerol solution and observed with an optical microscope (Nikon Eclipse E400). The genitalia slide was prepared in the following steps: The removed abdomen was placed in a 200 µl PCR tube filled with 10% solution of potassium hydroxide (KOH) and kept in heated water for about seven minutes. After rinsing in 70% ethanol solution, the abdomen was dissected by cutting its lateral side using fine insect pins. The genitalia were transferred to a cavity slide glass with 50% glycerol solution for observation. After the observation, the genitalia and abdomen were mounted in Euparal on cover glasses each glued to a piece of cardboard, and pinned with the specimens.

Photographs of adults were taken with digital camera (Canon EOS 7D), and composite images were produced using automontage software Combine ZM. These images were retouched using Photoshop 6.0 (Adobe Systems Inc.).

The larvae were preserved in 70% ethanol (Stehr 1987), and the dissected specimens were mounted in Euparal. Two larval specimens were dehydrated with absolute ethanol and sputter-coated with gold-palladium with a JEOL Ion Sputter JFC-1100 for examination with a scanning electron microscope (SEM). SEM photographs were taken using JSM-5600LV.

### Terminology, abbreviations and specimen deposition

Technical terms of genital structures follow Halstead (1980) and Lawrence et al. (2011) and larval morphology follows Stehr (1991), Thomas and Leschen (2010) and Lawrence et al. (2010).

The lectotype and the paralectotype designated herein are deposited in the Natural History Museum, London (BMNH). The other specimens examined in this paper are deposited in the Ehime University Museum, Matsuyama (EUMJ) and the Entomological Laboratory, Kyushu University, Fukuoka (ELKU).

## Results

### Description

#### Cryptamorpha sculptifrons

Reitter, 1889

http://species-id.net/wiki/Cryptamorpha_sculptifrons

Japanese name: Semaru-hoso-hiratamushi

**Figure 1.**
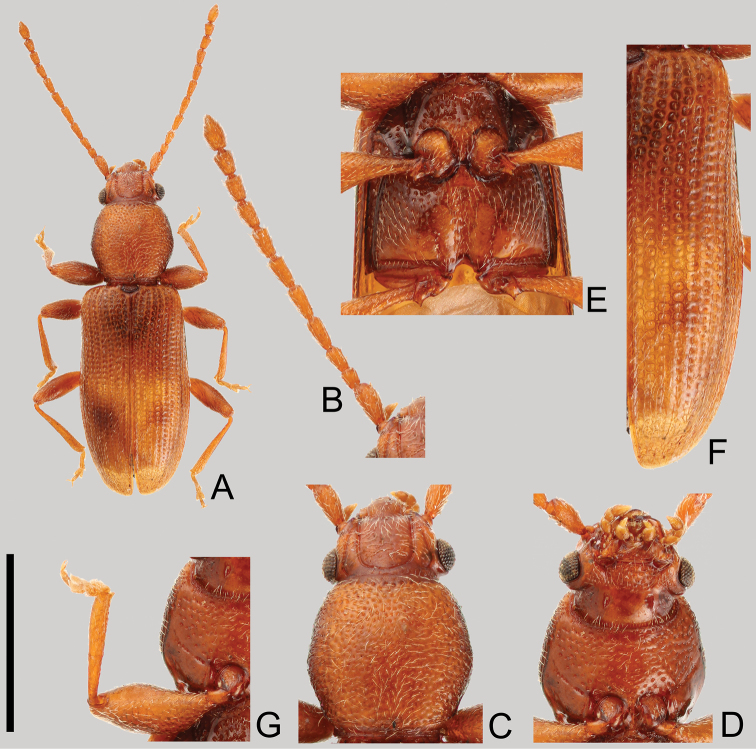
*Cryptamorpha sculptifrons* Reitter, 1889, lectotype, male. **A** Habitus, dorsal view **B** left antenna **C** head and pronotum, dorsal view **D** head and pronotum, ventral view **E** metaventrite **F** right elytron **G** right foreleg, ventral view. Scale: 2.0 mm for **A** and 1.0 mm for **B–G.**

Figs 1 [Fig F2] [Fig F3] [Fig F4] –5

Cryptamorpha sculptifrons Reitter, 1889: 320. – Grouvelle 1908: 474–476. – Grouvelle 1919: 46. – Hetschko 1930: 89. (catalogue) – Hisamatsu 1961: 2–3. – Kamiya 1961: 18, pl. 5. – Sasaji 1985: 205, fig. 36 in pl. 32. – Pal and Sen Gupta 1979: 77, 79, fig. 7. – Satô 1989: 377. – Halstead et al. 2007: 497. (catalogue) – Hirano 2009: 62. – Hirano 2010: 9–10.

##### Adults.

Body length from anterior margin of clypeus to apex of elytra measured along the median line: 3.69–4.14 mm (n=14).

**Coloration** (Fig. 1A). Surface brown, yellowish-brown in some lighter colored specimens. Elytra brown, sometimes slightly lighter than head and pronotum, with a variable round dark macula at each posterior half; elytra of lectotype with dark maculae. Antennae unicolorous, brown, as in head and pronotum.

**Head** (Fig. 1B, C and D). Triangular, width across eyes 0.76–0.86 mm. Temples immediately narrowed behind eyes, slightly incised at bases, with short setae except around eyes. Eyes relatively small, somewhat prominent, diameter less than half of length of head. Ventral surface with rough irregular punctuation except posterior half; dorsal surface with sparser punctuation. Antennal length 2.07–2.09 mm, 2nd antennomere short; 9th and 10th widened distally; covered with medium length semi-erect pubescence on each antennomere, 11th (apex) with short pubescence; approximate ratio of the length of each antennomere in lectotype as follows: 2.2: 1.0: 1.4: 1.7: 1.8: 1.7: 1.5: 1.5: 1.2: 1.2: 1.8 (Fig. 1B).

**Prothorax** (Fig. 1C and D). Rectangular, longer than wide, maximum width near middle, length 0.84–1.01 mm, width 0.89–0.95 mm in male (n=8) and 0.91–0.82 mm in female (n=5). Dorsal surface punctuated moderately densely except around posterior margin. Pubescence composed of many medium length setae on the dorsal side, and several relatively short setae on the ventral side. Each anterior angle with a few very small protuberances, with depressions around posterior angles of ventral surface. Procoxae without punctuation.

**Elytra** (Fig. 1F). Elongate, length 2.38–2.62 mm, combined width 1.22–1.43 mm. Punctures a little wider than interstices, scutellary striole composed of seven or eight punctures. Pubescence composed of semi-erect medium length setae.

**Legs** (Fig. 1E and G). Trochanter with a tooth on apical angle of inner margin. Profemora stout, maximum width near middle. Mesotibia curved inwards around apical 1/4.

**Figure 2. F2:**
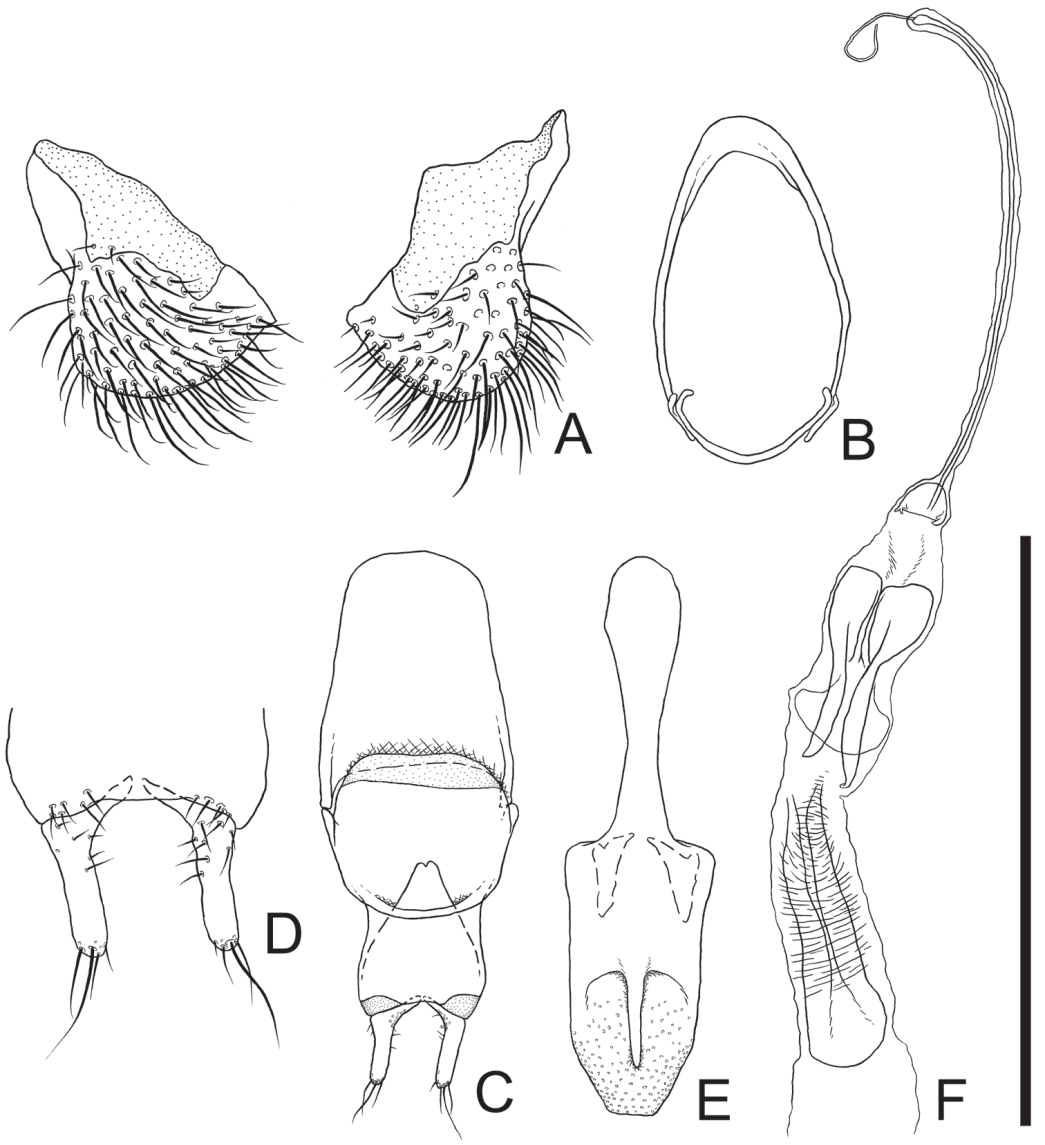
Male genital organs of *Cryptamorpha sculptifrons* Reitter, 1889, lectotype, male. **A** 8th sternite, ventral view **B** 9th sternite **C** phallobase, dorsal view **D** parameres, ventral view **E** penis, dorsal view **F** internal sac, dorsal view. Scale: 0.5 mm for **A**, **D** and 1.0 mm for **B**, **C**, **E**, **F.**

**Eighth and 9th sternites** (Fig. 2A and B). Eighth sternite (Fig. 2A) triangular, furcated near middle, each posterior half semicircular, covered with many medium length and some short setae, membranous around anterior portions. Ninth sternite (Fig. 2B) composed of two curved elements forming a ring: a short posterior semicircular strut strongly curved inwards at each end, connected to a long anterior element by a membrane; the long element strongly curved at middle, with a short dorsally orientated subapical process on each end, and the anterior half broadened.

**Aedeagus** (Fig. 2C–F). Parameres (Fig. 2C and D) L-shaped, relatively long, basal portions strongly curved inwards, inner dorsal margin of basal portion with several punctures, several medium length setae sparsely grouped around half of ventral surface, one or two long, a few medium length and a few short setae on each apex. Phallobase (Fig. 2C) rectangular, longer than wide, membranous around posterior angles, anterior margin of dorsal portion curled up at posterior 2/7, anterior angles protruding anterolaterally; each lateral portion strongly extended posteriorly, exposed from anterior margin of dorsal portion, connecting at posterior 1/3, membranous around anterior margin. Penis (Fig. 2E) elongate, posterior half relatively wide, somewhat flat, moderately punctuated on posterior 1/5, with blunt apex, and dorsal portion thinly extended posteriorly.

##### Sexual dimorphism

**Figure 3. F3:**
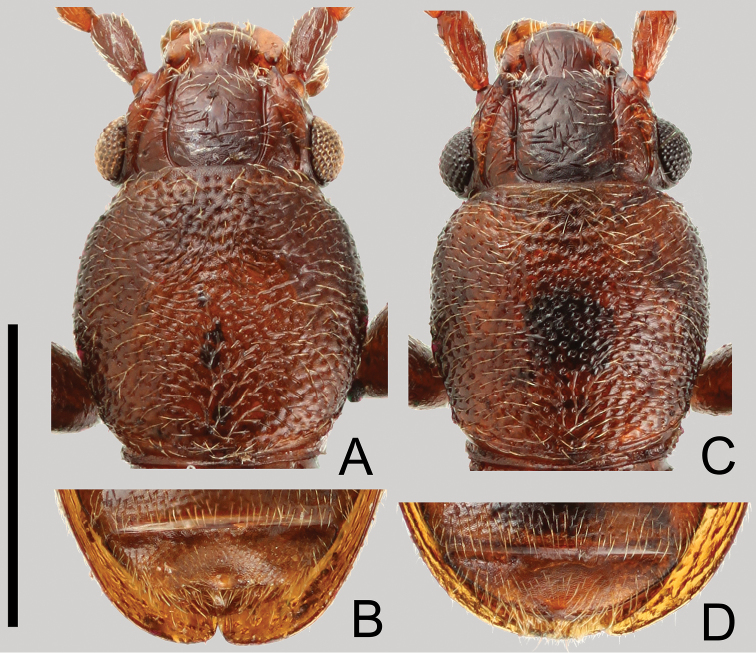
Sexual dimorphism of *Cryptamorpha sculptifrons* Reitter, 1889. **A**, **B** male **C**, **D** female. **A**, **C** Head and pronotum, dorsal view; **B**, **D** 7th sternite, ventral view. Scale: 1.0 mm.

(Fig. 3). Males and females are very similar. However, males can be distinguished from females by the wider pronotum which is comparatively expanded (Fig. 3A) and the strongly impressed central area of the 7th sternite with a small protrusion around middle of posterior margin (Fig. 3B).

##### Type series.

Lectotype here designated: male, Chûzenji, Nikkô City, Tochigi Prefecture, Japan, 19–24–VIII–1881, G. Lewis leg. (BMNH). Paralectotype: 1 male, same data as lectotype (BMNH).

##### Specimens examined.

JAPAN: [Gunma Pref.] 2 females, Hatomachi-Pass, Katashina Village, 29–VIII–1978, Y. Hori leg. (EUMJ). [Nagano Pref.] 1 female, Mt. Kisokomagatake, 6–VII–1960, Y. Kimura leg. (EUMJ). [Yamanashi Pref.] 1 male, Takizawa-Rindô, Narusawa Village, 1–VIII–2005, T. Kurihara leg. (EUMJ). [Gifu Pref.] 2 female, 5 male & 1 ex., Nigorigo-Onsen, Gero City, 14–15–VIII–2013, N. Tsuji leg. (ELKU).

##### Distribution.

JAPAN: Honshu; China, Bhutan, India (Pal and Sen Gupta 1979).

##### Remarks.

Syntypes of this species consisted of two specimens. We designate a brownish male specimen as lectotype, and another male yellowish-brown specimen as paralectotype, because the original description expressed their color as “testacea”, which means reddish brown color (Reitter 1889). Type specimens were collected from Chûzenji, Nikkô City, Tochigi. This species was collected from relatively high altitudes, and there is no specimen collected from western Japan.

##### Mature larva.

Head capsule width of mature larvae: 0.90–0.96 mm (n=27).

**Coloration.** Body white to yellowish white. First antennomere, posterior 4/5 of 2nd and anterior half of 3rd somewhat darkened. Frontal arm white. Most setae, base and posterior 2/7 of urogomphi brownish white.

**Figure 4. F4:**
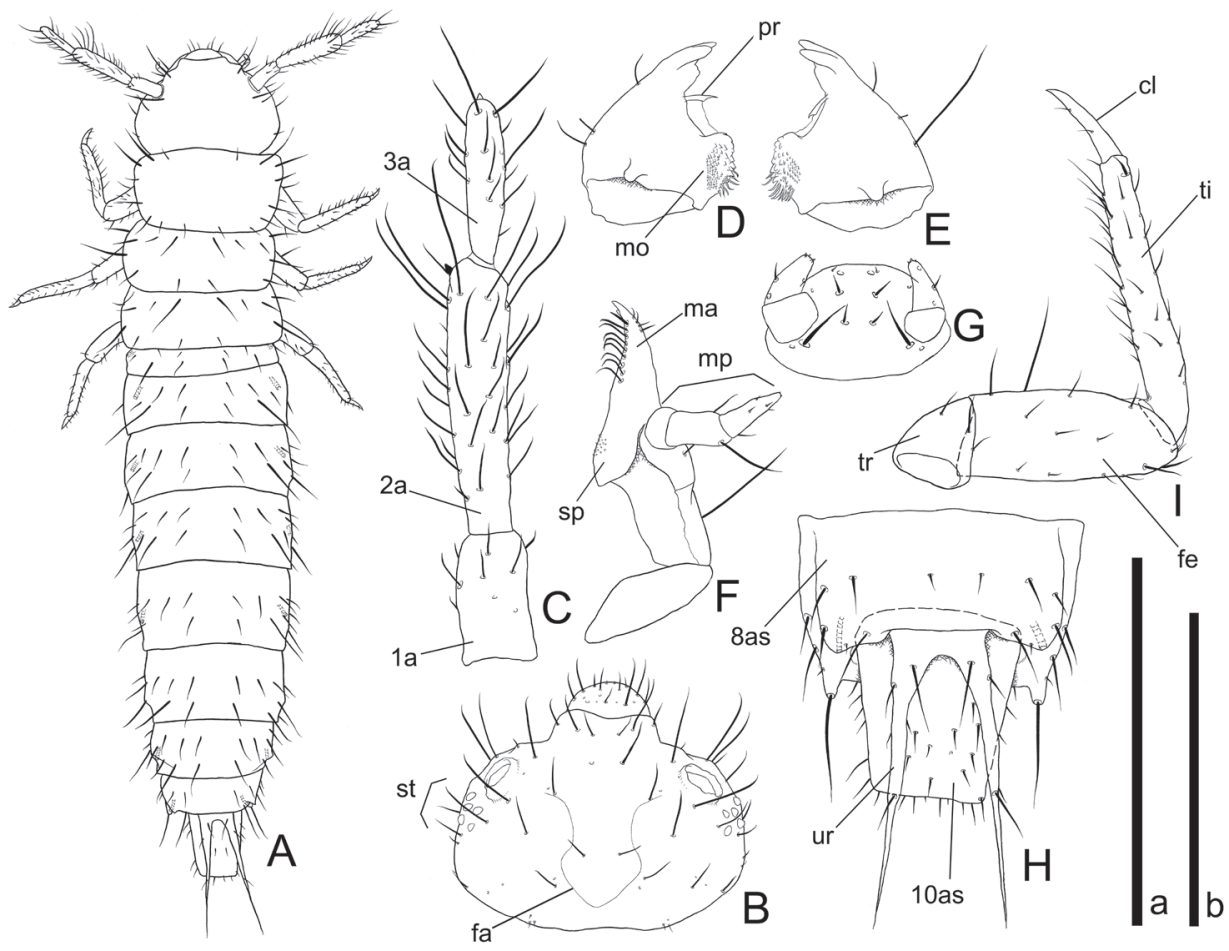
Mature larval morphology of *Cryptamorpha sculptifrons* Reitter, 1889. **A** Habitus, dorsal view **B** head, dorsal view; **C** right antenna, dorsal view **D** right mandible, ventral view **E** left mandible, ventral view **F** right maxilla, dorsal view **G** labium, ventral view **H** 8th to 10th abdominal segments, dorsal view **I** right foreleg, dorsal view. Abbreviations: cl–claw; fa–frontal arm; fe–femur; ma–mala; mo–mola; mp–maxillary palpus; pr–prostheca; sp–stipe; st–stemmata; ti–tibiotarsus; tr–trochanter; ur–urogomphus; 1a–1st antennomere; 2a–2nd antennomere; 3a–3rd antennomere; 8as–8th abdominal segment; 10as–10th abdominal segment. Scale: a for **A–H**, 2.0 mm for **A**, 1.0 mm for **B**, **H**, 0.5 mm for **C–F** and 0.3 mm for **G**; b for **I**, 0.5 mm.

**Figure 5.**
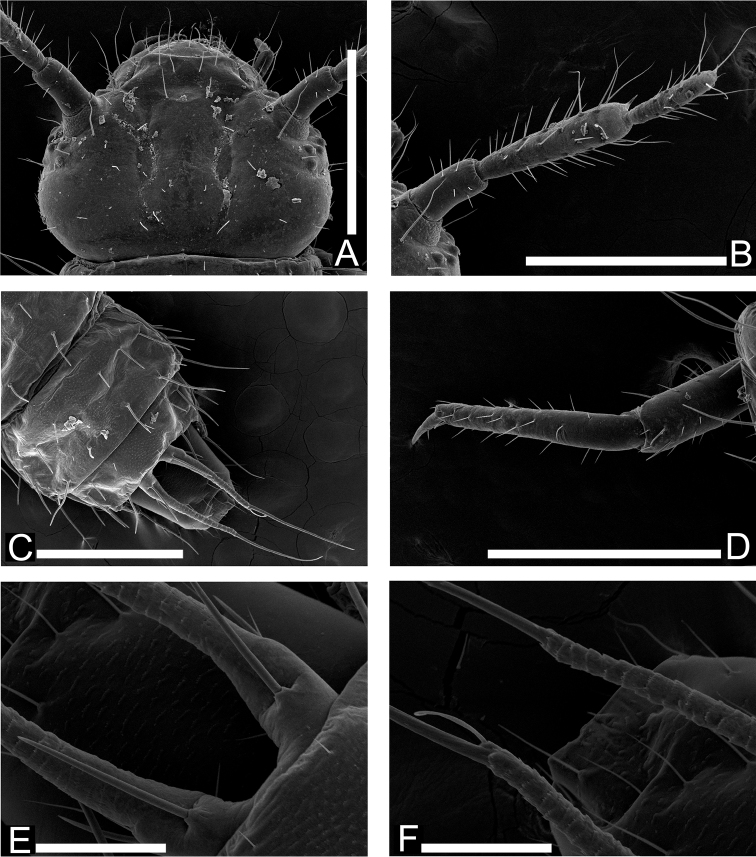
Mature larval morphology of *Cryptamorpha sculptifrons* Reitter, 1889. **A** Head, dorsal view **B** right antenna, dorsal view **C** 8th to 10th abdominal segments, dorsal view **D** left foreleg, dorsolateral view **E**, **F** urogomphi, dorsal view, basal portion (**E**) and middle portion (**F**). Scale: 0.5 mm for **A–D** and 0.1 mm for **E**, **F.**

**Head** (Figs 4A–G, 5A and B). Rectangular, covered with medium length and long setae and several short setae. Frontal arm U-shaped (Figs 4B and 5A). Antenna (Figs 4C and 5B) moderately long; 1st antennomere stout but relatively long, apical half covered with relatively short setae; 2nd subparallel, relatively thick, twice as long as 1st, covered with many variable length setae, a short and thick conical sensorium present near apex of inner margin; 3rd less than 1.5 times as long as 1st, covered with many short and a few long setae, a stout and short seta present on apex. Mandibles (Fig. 4D and E) triangular with two acute and one blunt teeth around each apex; a prostheca on anterior 1/3 of each inner margin, anterior angles strongly pointed; molae on bases of inner margins enlarged in anterolateral direction with many minute spines; several fine spines around inner margins of posterior half; a few medium length setae and a long seta on each outer margin. Maxilla (Fig. 4F) oblong; mala diverging at apex, with several short setae on outer margin near apex, inner margin with relatively thick and long setae in a row of which the most apical seta spiniform; stipes with many moderately dense minute spines around inner margin of central dorsal areas; maxillary palpus 3-segmented; 1st spherical and short; 2nd more than twice as long as 1st, with a few long setae on apical portion; 3rd almost as long as 2nd with a few minute spines on apex and some short setae. Labium (Fig. 4G) round, covered with several short setae and a pair of relatively long setae; palpus two segmented, covered with a few short setae, a few very short spines on each apex. Six stemmata on each lateral portion (Fig. 5A).

**Thorax and abdomen** (Figs 4A, H, 5C, E and F). The shape easily deformed according to their posture. Thoraxes covered with short and medium length setae, a pair of relatively long setae on middle lateral margins, two relatively long setae on anterior angles of prothorax. Abdomen 10-segmented; 1st abdominal segment short, 2nd to 8th rectangular, wider than long, posterior angles of 8th strongly protruding, covered with some variably sized setae on each segment, a long seta on apex of each posterior angle of 8th (Fig. 4H), 9th concealed but urogomphi exposed from under posterior margin of 8th abdominal tergite, less than twice as long as 10th, deeply emarginate around base, a few medium length setae around base and a relatively short seta on middle of each branch, anterior half of each branch scaly (Figs 4H, 5C, E and F), 10th longer than wide, covered with relatively short setae.

**Legs** (Figs 4I and 5D). Elongate. Trochanter triangular, covered with a few medium length setae; femur relatively thick, covered with short and medium length setae and one or two long setae around inner margin; tibiotarsus elongate, curved strongly inwards around base, covered with many medium length setae and a relatively stout seta around apex; claw elongate, weakly curved inwards, apex pointed, with two short setae.

##### Specimens examined.

27 exs. (two specimens were examined with slide preparation, and two specimens with a SEM), Nigorigo-Onsen, Gero City, Gifu Prefecture, Japan, 14–15–VIII–2013, N. Tsuji leg. (ELKU).

##### Biology.

According to Mr. Tsuji (pers. comm.), the mature larvae were collected from dead leaves of Southern Japanese hemlock *Tsuga sieboldii* with their adults.

##### Identification.

The examined larvae were collected with adult individuals of *Cryptamorpha sculptifrons*. They were identified as a member of the subfamily Brontinae from the generic key to the known larvae of some cucujoid families (Thomas 1988). The larvae of the genus *Cryptamorpha* can be distinguished from those of the genus *Psammoecus* by the presence of urogomphi on the 9th abdominal segment (Pal 1985 and Hayashi 1992). In addition, *Cryptamorpha sculptifrons* is the only *Cryptamorpha* species distributed in mainland Japan. On the basis of this data, we identified the collected larvae as *Cryptamorpha sculptifrons*.

## Discussion

### Distribution of *Cryptamorpha sculptifrons*

Grouvelle (1908) described two varieties, *Cryptamorpha sculptifrons* var. *punctifrons* from India and *Cryptamorpha sculptifrons* var. *opacifrons* from China (Yun-nan) and India. He noted that these varieties differed from Japanese *Cryptamorpha sculptifrons* by their body shape and the shapes of the 1st to 3rd antennomeres, and suggested that reexamination of more numerous individuals of var. *punctifrons* might justify the separation of this variety from Japanese *Cryptamorpha sculptifrons*. Pal and Sen Gupta (1979) recorded this species based on specimens from Bhutan similar to var. *punctifrons* with an illustration of their male genitalia. However, the male genital structure of the Japanese species (Fig. 2C-E) differs from these by the longer setae on the apex of the parameres and the shape of the penis, which has a roundly prominent apex in Bhutanese specimens. Thus, var. *punctifrons* seems to represent a distinct species, and reports of the occurrence of *Cryptamorpha sculptifrons* in India and Bhutan are considered unreliable. Further examination of the specimens studied by Grouvelle (1908) and specimens of the genus *Cryptamorpha* collected in South East Asia are required.

### Taxonomic importance of larval morphology

Silvanid classification is in its infancy, and there is only one preliminary phylogenetic analysis for this family, treating 20 genera, based on 15 characters, by Thomas and Nearns (2008). In the analysis of Thomas and Nearns (2008), no more than three larval characters were used, and they were known in about half of the species treated. In the genus *Cryptamorpha*, larval morphology is known only for two species, *Cryptamorpha desjardinsi* and *Cryptamorpha brevicornis* (White, 1846). The former was included in a taxonomic key of some Cucujoid families and the latter was described by Hudson (1924) without describing the mouth parts. Thus, our paper provides the first description of the mouth parts of a *Cryptamorpha* larva.

In the key to known larvae of America North of Mexico by Thomas (1988), the genus *Cryptamorpha* was distinguished from members of the tribe Brontini by the presence of urogomphi that are shorter than the 10th abdominal segment and the absence of strongly protruded posterior angles of the 8th abdominal segment. However, *Cryptamorpha sculptifrons* (not occurring in the New World) possesses urogomphi that are longer than the 10th abdominal segment and strongly protruding posterior angles of the 8th abdominal segment, though they are not protruding as strongly as those of the Brontini (Figs 4H, 5C). Including further information on the morphology of this species, larvae of *Cryptamorpha* can be distinguished from members of the tribe Brontini by having relatively thick antennae and the 3rd antennomere which is less than 3/4 of the length of the 2nd in the larval morphology.

Accumulation of detailed descriptions of the immature stages of more Silvanid taxa would be required for more accurate inferences on phylogenetic relationships and the completion of a more correct taxonomic key to larvae.

## Supplementary Material

XML Treatment for Cryptamorpha sculptifrons

## References

[B1] Brown STJ Marris JWM Leschen RAB (2012) Review of New Zealand *Cryptamorpha* (Coleoptera: Silvanidae), with a description of a new species from the three kings islands. New Zealand Entomologist 35: 29–38. doi: 10.1080/00779962.2012.649706

[B2] Grouvelle A (1908) Coléoptères de la région Indienne. Rhysodidae, Trogositidae, Nitidulidae, Colydiidae, Cucujidae. Annales de la Société Entomologique de France 77: 315–495

[B3] Grouvelle A (1919) Descriptions d’espèces nouvelles du genre Cryptamorpha Woll In: Mèmoires Entomologiques. Études sur les coléoptères. II Société Entomologiques de France, Paris, 39–46

[B4] Halstead DGH (1980) A revision of the genus *Oryzaephilus* Ganglbauer, including descriptions of related genera (Coleoptera: Silvanidae). Zoological Journal of the Linnean Society 69: 271–374. doi: 10.1111/j.1096-3642.1980.tb01126.x

[B5] Halstead DGH Löbl I Jelínek J (2007) Silvanidae. In: Löbl I Smetana A (Eds) Catalogue of Palaearctic Coleoptera. Vol. 4. Elateroidea – Derodontoidea – Bostrichoidea – Lymexyloidea – Cleroidea – Cucujoidea. Apollo Books, Stenstrup, 496–501

[B6] Hayashi N (1992) Illustritions for identification of larvae of the superfamily Cucujoidea (Coleoptera) found in mouldy stored foods in Japan. House and Household Insect Pests 14(2): 102–131 [In Japanese, with English title]

[B7] Hetschko A (1930) Fam. Cucujidae In: Coleopterorum Catalogus Pars 109 W. Junk, Berlin, 1–90

[B8] Hirano Y (2009) Notes on Japanese Silvanidae (Nihonsan hosohiratamushi-ka ni tsuite). Kanagawa-chûhô 168: 57–83 [In Japanese]

[B9] Hirano Y (2010) Cucujoidea of Japan Vol. 2 Silvanidae, Byturidae, Biphyllidae. Roppon-Ashi Entomological Books, Tokyo, 61 pp. [In Japanese, with English title]

[B10] Hisamatsu S (1961) Illustrations of the small beetles in Japan [III]. Ageha 9: 1–5 [In Japanese, with English title]

[B11] Hudson GV (1924) Illustrated life histories of New Zealand insects. Transactions and Proceedings of the New Zealand Institute 55: 341–343

[B12] Kamiya H (1961) How to identify flat bark beetle (Nihonsan hiratamushi no miwake-kata). Tsukushi no Konchu 6: 15–18 [In Japanese]

[B13] Lawrence JF Beutel RG Leschen RAB Ślipiński A (2010) Glossary of morphological terms. In: Leschen RAB Beutel RG Lawrence JF (Eds) Handbook of Zoology, Coleoptera, Beetles, Vol. 2: Morphology and Systematics (Elateroidea, Bostrichiformia, Cucujiformia partim). Walter de Gruyter, Berlin New York, 9–20

[B14] Lawrence JF Ślipiński A Seago AE Thayer MK Newton AF Marvaldi AE (2011) Phylogeny of the Coleoptera based on morphological characters of adults and larvae. Annales Zoologici (Warszawa) 61: 1–217. doi: 10.3161/000345411X576725

[B15] Leschen RAB Lawrence JF Ślipiński SA (2005) Classification of basal Cucujoidea (Coleoptera: Polyphaga): cladistic analysis, keys and review of new families. Invertebrate Systematics 19: 17–73. doi: 10.1071/IS04007

[B16] Murakami O Washitani I (2002) Handbook of alien species in Japan. Chijin Shokan, Tokyo, 390 pp. [In Japanese, with English title]

[B17] Pal TK (1985) A revision of Indian *Psammoecus* Latreille (Coleoptera: Silvanidae). Records of the Zoological Survey of India 71: 1–54

[B18] Pal TK Sen Gupta T (1979) Ergebnisse der Bhutan-Expedition 1972 des Naturhistorischen Museums in Basel. Coleoptera: Fam. Silvanidae. Entomologica Basiliensia 4: 69–82

[B19] Reitter E (1889) Verzeichniss der Cucujiden Japans mit beschreibungen neuer arten. Wiener Entomologische Zeitung 8: 299–304

[B20] Sasaji H (1985) Silvanidae In: Kurosawa Y Hisamatsu S Sasaji H (Eds) The Coleoptera of Japan in Color Vol. III Hoikusha Publishing, Osaka, 202–205 (incl. pl.32) [In Japanese, with English title]

[B21] Satô M (1989) Coleoptera. In: Hirashima Y (Ed) A Check List of Japanese Insects I Entomological Laboratory, Faculty of Agriculture, Kyushu University, Fukuoka, 197–538 [In Japanese]

[B22] Stehr FW (1987) Immature Insects. Kendall / Hunt Publ. Co., Dubuque, Iowa, 974 pp.

[B23] Stehr FW (1991) Techniques for Collecting, Rearing, Preserving, and Studying Immature Insects. In: Stehr FW (Ed) Immature Insects. Volume 2 Kendall / Hunt Publ. Co., Dubuque, Iowa, 7–18

[B24] Thomas MC (1988) Generic key to the known larvae of the Cucujidae, Passandridae, and Silvanidae of America north of Mexico (Coleoptera). Insecta Mundi 2: 81–89 http://digitalcommons.unl.edu/insectamundi/496

[B25] Thomas MC (2002) Silvanidae Kirby 1837. In: Arnett RH Thomas MC Skelley PE Frank JH (Eds) American beetles. Vol. 2. Polyphaga: Scarabaeoidea through Curculionoidea. CRC Press, Boca Raton, 322–326

[B26] Thomas MC Leschen RAB (2010) Silvanidae. In: Leschen RAB Beutel RG Lawrence JF (Eds) Handbook of Zoology, Coleoptera, Beetles, Vol. 2: Morphology and Systematics (Elateroidea, Bostrichiformia, Cucujiformia partim). Walter de Gruyter, Berlin New York, 346–350

[B27] Thomas MC Nearns EH (2008) A new genus of telephanine Silvanidae (Coleoptera: Cucujoidea), with a diagnosis of the tribe and key to genera. Insecta Mundi 0048: 1–14 http://digitalcommons.unl.edu/insectamundi/576

[B28] Yoshida T Hirowatari T (2013) A new species of the genus *Psammoecus* (Coleoptera, Silvanidae) from the Nansei Islands, Japan. Japanese Journal of Systematic Entomology 19: 85–90

[B29] Yoshida T Hirowatari T (2014) A revision of Japanese species of the genus *Psammoecus* Latreille (Coleoptera, Silvanidae). ZooKeys 403: 15–45. doi: 10.3897/zookeys.403.7145 24843265 10.3897/zookeys.403.7145 PMC4023238

